# Biomarkers of IgA vasculitis nephritis in children

**DOI:** 10.1371/journal.pone.0188718

**Published:** 2017-11-30

**Authors:** Evangeline Pillebout, Agnès Jamin, Hamza Ayari, Pierre Housset, Melissa Pierre, Virginia Sauvaget, Denis Viglietti, Georges Deschenes, Renato C. Monteiro, Laureline Berthelot, for the HSPrognosis group

**Affiliations:** 1 INSERM 1149, Center of Research on Inflammation (CRI), Paris, France; 2 Inflamex, Laboratory of Excellence, Bichat Medical Faculty, Paris, France; 3 University Paris Diderot, Sorbonne Paris Cité, Paris, France; 4 CNRS ERL8252, Paris, France; 5 Department of nephrology, Saint-Louis Hospital, AP-HP, Paris, France; 6 Department of Pediatric Nephrology, Robert Debré Hospital, AP-HP, DHU Fire, Paris, France; 7 Department of Immunology, Bichat Hospital, AP-HP, DHU Fire, Paris, France; 8 Centre de Recherche en Transplantation et Immunologie (CRTI), UMR 1064, INSERM, Université de Nantes, Nantes, France; University of Utah School of Medicine, UNITED STATES

## Abstract

Henoch–Schönlein purpura is a systemic vasculitis characterized by IgA deposits, which target the skin, joints, and kidneys, among other organs. In children, prognosis is often good but little is known about biomarkers of pediatric nephritis. We hypothesized that biological markers, including cytokines, immunoglobulins, IgA-immune complexes, IgA glycosylation and neutrophil gelatinase-associated lipocalin (NGAL), may discriminate IgA vasculitis (IgAV) pediatric patients with renal involvement from those without renal involvement. Fifty children at the time of IgAV rash between 2010 and 2015 were prospectively enrolled and compared to 21 controls. All patients were assessed for clinical and biological parameters at the time of diagnosis, including the levels of cytokines, immunoglobulins, immune complexes, IgA glycosylation and NGAL in serum and urine. Among IgAV patients, 33 patients exhibited nephritis (IgAV-N) and 17 children were without nephritis (IgAV-woN). The serum level of galactose-deficient (Gd)-IgA1 (p<0.01) and the urinary concentrations of IgA, IgG, IgM, IL-6, IL-8, IL-10, IgA-IgG complexes and IgA-sCD89 complexes (p<0.001 for all) were higher in the IgAV-N patients than in the IgAV-woN patients. Among those markers, urinary IgA and IgM had the highest AUC (0.86 and 0.87 respectively, p<0.0001). This prospective cohort study furthers our understanding of the pathophysiology of IgAV. We identified biomarkers that are able to distinguish patients initially with or without nephritis. To conclude, serum Gd-IgA1 and urinary IgA, IgG, IgM, IL-6, IL-8, IL-10, and IgA-IgG and IgA-sCD89 complexes could identify IgAV pediatric patients with renal involvement at the time of diagnosis.

## Introduction

In children, IgA vasculitis (IgAV) or Henoch-Schönlein purpura is a common systemic vasculitis affecting the small vessels of the skin and other organs, particularly the kidneys, joints and intestines [1]. Most of the cases are self-limiting but nephritis occurs in 20 to 54% of children leading to complications [2–5]. The natural history of nephritis ranges from persistent asymptomatic microscopic hematuria to progressive kidney failure, with an estimated long-term risk of end-stage renal failure of up to 15% in children [3]. Non-invasive biomarkers of nephritis are specifically required for children to avoid kidney biopsies.

IgAV is considered an inflammatory disease involving IgA immune complexes. The pathological mechanisms of organ-specific IgA deposition remain elusive. IgAV nephritis seems to share pathological mechanisms with IgA nephropathy. Correlations between immune parameters and organ injury have been explored suggesting a Th1/Th2 imbalance in Chinese children [6]. Moreover, the galactose deficiency of circulating IgA1 is associated to with nephritis as described in IgA nephropathy [7,8].

However, studies on IgAV pediatric cohorts are rare, particularly those exploring inflammatory mechanisms. Therefore, we assessed the levels of cytokines, immunoglobulins, IgA-immune complexes, IgA glycosylation and neutrophil gelatinase-associated lipocalin (NGAL) in blood and urine in a well characterized population of 50 children with IgAV to discriminate pediatric patients with renal involvement from those without renal involvement.

## Materials and methods

### Pediatric population

This cohort of children is part of a French national, multicenter, prospective study (HSPrognosis study) which was conducted between April 2010 and May 2015. Adult patients with IgAV (n = 85) were previously described [9]. The local ethical committee of Assistance Publique–Hôpitaux de Paris (AP-PH, approval number 10.649bis) approved this study, and written informed consent was obtained from the parents of all the included children before any study-related procedure was performed. Patients were enrolled at the time of skin rash appearance in the pediatric and emergency departments of the hospitals (most patients were recruited in Paris’ region except 2 from the South of France). Biological samples were concomitantly collected at the skin rash. Pediatric controls (healthy controls who underwent a systemic familial investigation and pediatric cases of a single kidney) were enrolled in a single center (R. Debré’s Hospital, Paris, France), and controls with hematuria or proteinuria >0.5 g/g were excluded. The inclusion criterion was a clinical diagnosis of IgAV (EULAR/PRINTO/PRES criteria [10]). The exclusion criteria were the following: (i) absence of skin lesions; (ii) treatment with immunosuppressive drugs or steroids during the 2 weeks prior to skin rash appearance; (iii) thrombocytopenia (platelet number <100,000/mm^3^); and (iv) absence of consent.

Renal involvement was defined as the presence of hematuria (determined by dipstick or a red cell count >5/mm^3^) and/or a proteinuria/creatininuria ratio ≥0.5 g/g and/or an eGFR <60 mL/min in children (determined using the Schwartz formula) [11].

A renal biopsy was performed if indicated by clinical examination and biological tests, because of hematuria and a ratio of proteinuria/creatininuria (PCR) greater than 0.5 g/g and/or renal failure. All renal biopsies were independently examined by two pathologists in blinded conditions. On immunofluorescence, the predominance of mesangial IgA among glomerular immune deposits was examined. At least ten glomeruli had to be present on the biopsy. We semi-quantitatively evaluated: (1) endocapillary lesions and their focal or diffuse distribution; (2) extracapillary proliferation, graded according to the number of glomeruli involved; and (3) interstitial fibrosis (area percentage). The proportions of glomeruli involved by crescents, fibrinoid necrosis, and global sclerosis were also recorded. All biopsies were then classified according to the ISKDC classification standard: Type I, minimal glomerular abnormalities; Type II, mesangial proliferation without crescents; Type III, focal segmental (IIIa) or diffuse (IIIb) mesangial proliferation with<50% crescents; Type IV, mesangial proliferation with 50–70% crescents; Type V, mesangial proliferation with>75% crescents; and Type VI, membrano-proliferative-like lesions.

### Immunoglobulin, cytokine and NGAL measurements

The concentrations of IgA, IgM and IgG were determined in sera by nephelometry at the Immunology Department of Bichat Hospital. The levels of IgD and IgE were detected in sera, and all other immunoglobulin concentrations in urine samples were assessed by ELISA using Bethyl kits (Bethyl Laboratories, Montgomery, Texas, USA). Cytokine Binding Assay kits (BD Biosciences, Le Pont de Claix, France) were used to determine the concentrations of cytokines, including interleukin (IL)-1β, IL-6, IL-8, IL-10, IL-12p70 and tumor necrosis factor (TNF-α) in plasma and urine, and bead fluorescence was analyzed on a FACSCanto II cytometer (BD Biosciences, Le Pont de Claix, France). The NGAL concentration was determined using ELISA kits (Bioporto, Hellrup, Denmark).

### ELISA for IgA1 glycosylation

IgA1 glycosylation was assessed by a lectin ELISA using Helix aspersa agglutinin (HAA). In brief, plates were coated with anti-IgA antibody (1/500 in PBS, Bethyl Laboratories Montgomery, Texas, USA) overnight at 4°C, and the blocking solution (PBS, bovine serum albumin 2%) was added and allowed to stand for 8 h at 4°C. Serum samples were diluted to 10 μg/mL for IgA and incubated overnight at 4°C. To remove sialic acids, neuraminidase was added and incubated for 3 h at 37°C, followed by the addition of biotinylated Helix aspersa agglutinin (HAA, Sigma-Aldrich, Saint-Louis, Missouri, USA) and an additional 3-h incubation at 37°C. The detection of HAA binding was performed using streptavidin coupled with alkaline phosphatase (1/10000, Jackson Immuno Research Laboratories, West Grove, PA, USA) and substrate (SIGMAFAST p-nitrophenyl phosphate tablets; Sigma-Aldrich, Saint-Louis, Missouri, USA). The plates were read at 405 nm using an Infinite M200 microplate reader (Tecan, Manndorf, Switzerland). The positive control (i.e., purified deglycosylated IgA1) corresponded to 100% binding.

### IgA complex detection

For the IgA complex measurements, ELISA was performed as follows: coating with anti-IgA antibody (1/500 in PBS, Bethyl Laboratories, Montgomery, Texas, USA) and with A3 anti-CD89 (5 μg/mL, produced at laboratory) for IgA/IgG and IgA-sCD89, respectively, overnight at 4°C, followed by blocking for 8 h at 4°C. Polyethylene glycol-precipitated serum or urine samples were incubated overnight at 1/10. Revelation was performed using anti-IgG (1/5000, Southern Biotech, Birmingham, Alabama, USA) or anti-IgA (1/2000, BD Biosciences Le Pont de Claix, France) coupled with alkaline phosphatase. The positive control (i.e., a control serum with high levels of complexes) corresponded to 100% binding, and all samples were compared to this positive control.

### CD89 expression on blood myeloid cells

Blood samples (50 μL) were labeled with antibodies (BD Biosciences) as follows: anti-CD11b coupled with Pacific Blue, anti-CD15 coupled with allophycocyanin (APC), anti-CD14 coupled with APC-H7, and anti-CD89 coupled with phycoerythrin (PE) or isotype-PE for 20 min at room temperature. Then, red blood cells were lysed using BD lysing solution (BD Biosciences, Le Pont de Claix, France) for 10 min and then washed twice with a PBS solution of 2% bovine serum albumin and 0.01% azide. The samples were analyzed using a FACSCanto II system (BD Biosciences, Le Pont de Claix, France) and FlowJo software.

### Statistical analysis

Median (interquartile range IQR) values and frequencies are provided for the description of continuous and categorical variables, respectively. The Shapiro-Wilk test was used to test the normality of the data distribution. The mean values and proportions were compared using Mann-Whitney and Fisher’s exact tests, respectively. Aberrant values for biological variables were determined by a Dixon test and excluded. Spearman's rank correlation coefficient was used to assess the correlation between two continuous variables. For each biomarker, we constructed an ROC curve and calculated the Area Under the Curve (AUC) to evaluate its predictive accuracy to discriminate renal involvement and outcome. A non-parametric method was used to calculate the confidence interval (CI). All tests were two-sided, and values of p<0.05 were considered statistically significant. All statistical analyses were performed with GraphPad Prism version 7 (GraphPad Software).

## Results

### Characteristics of the pediatric cohort

Fifty children with IgAV and 21 pediatric controls (sex and age matched) were prospectively recruited to compose the study population. The demographic and baseline disease characteristics are presented in Table 1. The median age was 7 (6–9) years for children. Male gender represented 66.0% of the overall study population. Seventeen (34.0%) patients did not exhibit renal involvement (IgAV-woN group), and 33 (66.0%) exhibited nephritis (IgAV-N group) as defined by clinical parameters (hematuria and-/or proteinuria and/or eGFR<60 mL/min). For 23 children, the renal involvement was confirmed by a kidney biopsy. Nearly 93.9% of pediatric IgAV-N patients exhibited hematuria and 78.8% proteinuria over 1 g/g. Renal failure was less prevalent in children than in adults (3.0% of pediatric IgAV-N patients compared with 40.0% of adult IgAV-N patients (HSPrognosis study group, p<0.0001). The median proteinuria creatininuria ratio (PCR) was 2.5 (0.54–6.20) g/g and the median serum creatinine level 41.0 (34.5–57.0) μmol/L (for mg/dl conversion, divide by factor 88.4) for the pediatric IgAV-N group. The most frequent lesion in renal biopsies was diffuse mesangial proliferation with less than 50% crescent (class IIIb, 52.2%, Table 2). Only 3 patients had more than 50% crescents (two patients were class IV and one patient class V). None of the patients had global sclerosis and interstitial fibrosis was rare. After one year, 2 children were lost to follow-up. As described in other cohorts, the renal prognosis was good. No children exhibited de novo renal involvement or renal failure. The median proteinuria/creatininuria ratio only reached 0.12 (0.06–0.23) g/g for the IgAV-N group after one year. Only one (2.0%) patient maintained PCR >1 g/g, but the serum creatinine concentration was low (49.8 μmol/L). Concerning treatments, most of the patients received steroids (65.5%), 22 (45.8%) received Angiotensin Converting Enzyme inhibitors (ACEi) or Angiotensin Receptor Blockers (ARB), 15 (31.2%) patients received no treatment, 4 (8.3%) received cyclophosphamide and 1 (2.0%) rituximab. There was no correlation between histological classification and proteinuria after one year of follow-up (Table 2) or other clinical parameters.

**Table 1:** Patient characteristics.

	Controls	IgAV-woN	IgAV-N
**Number** (percent)	21	17 (34.0%)	33 (66.0%)
**Age** (years): median (IQR)	8 (7–11)	6 (5–8)	8 (7–10) ^**a**^
**Male gender**: number (percent)	13 (61.9%)	12 (70.5%)	20 (60.6%)
**Skin involvement**: number (percent)	0	17 (100%)	33 (100%)
**Joint involvement**: number (percent)	0	12 (70.6%)	26 (78.7%)
**Gastrointestinal involvement**: number (percent)	0	7 (41.2%)	20 (60.6%)
**Renal involvement**: number (percent)	0	0	33 (100%) ^**b**^^,^^**c**^
**Hematuria**: number (percent)	0	0	31 (93.9%) ^**b**^^,^^**c**^
**PCR ≥ 0.5 g/g**: number (percent)	0	0	26 (78.8%) ^**b**^^,^^**c**^
**PCR** (g/g): median (IQR)	0.05 (0.04–0.07)	0.05 (0.04–0.09)	2.5 (0.54–6.20) ^**a**^^,^^**d**^
**PCR group**			
**0.5–1 g/g**: number (percent)	0	0	4 (12.1%)
**1–3 g/g**: number (percent)	0	0	9 (27.3%) ^**a**^^,^ ^**d**^
**≥ 3 g/g**: number (percent)	0	0	13 (39.4%) ^**a**^^,^ ^**d**^
**eGFR** (mL/min/1.73m^2^): median (IQR)	137.0 (102.5–169.3)	165.1 (139.3–206.2)	152.1 (118.7–180.1)
**eGFR group**			
**≥ 90 mL/min/1.73m**^**2**^: number (percent)	20 (95.2%)	16 (94.1%)	31 (93.9%) ^**b**^
**60–89 mL/min/1.73m**^**2**^: number (percent)	1 (4.8%)	0	1 (3.0%) ^**b**^
**30–59 mL/min/1.73m**^**2**^: number (percent)	0	1 (5.9%)	1 (3.0%)
**15–29 mL/min/1.73m**^**2**^: number (percent)	0	0	0
**< 15 mL/min/1.73m**^**2**^: number (percent)	0	0	0
**Renal failure (eGFR < 60)**: number (percent)	0	0	1 (3.0%) ^**d**^
**Serum creatinine** (μmol/L): median (IQR)	41.0 (31.7–66.0)	36.0 (29.0–43.8)	41.0 (34.5–57.0) ^**d**^

eGFR was calculated the Schwartz formula for children.

Conversion factor for serum creatinine in mg/dL to μmol/L, x 88.4.

The Mann-Whitney U test was used for median comparisons and Fisher exact test for categorical comparisons:

^**a**^ IgAV-woN vs IgAV-N, P<0.05

^**b**^ controls vs IgAV-N, P<0.0001

^**c**^ IgAV-woN vs IgAV-N, P<0.0001

^**d**^ IgAV-woN vs IgAV-N, P<0.01.

Abbreviations: IgAV-woN = IgAV patients without nephritis. IgAV-N = IgAV patients with nephritis. IQR = Interquartile range. PCR = proteinuria creatininuria ratio. eGFR = estimated glomerular filtration rate.

**Table 2:** Histological class from renal biopsies.

Histological Class	II	IIIa	IIIb	IV	V
**Number (percent)**	5 (21.7%)	3 (13.0%)	12 (52.2%)	2 (8.7%)	1 (4.3%)
**Age (years): median (IQR)**	9.0 (6.5–12.5)	4.0 (4.0–8.0)	8.0 (7.3–9.0)	12.0 (10.0–14.0)	7.0
**Male gender: number (percent)**	4 (80.0%)	2 (66.7%)	5 (41.6%)	2 (100%)	1 (100%)
**PCR (g/g) at day 1: median (IQR)**	0.55 (0.29–2.48)	3.12 (1.61–15.5)	5.55 (2.68–8.85)	2.35 (1.12–3.58)	5.5
**PCR (g/g) after one year**	0.24 (0.06–0.50)	0.22 (0.18–0.28)	0.10 (0.07–0.22)	1.61 (0.07–3.16)	0.07

Abbreviations: IQR = interquartile range. PCR = proteinuria creatininuria ratio.

### Immunoglobulins

The concentrations of immunoglobulins were measured in the serum and urine of children, collected at the time of the rash, before the onset of any treatment. The IgA and IgE serum concentrations of all IgAV patients were higher than those of the controls (p<0.0001), with no differences between IgAV-woN and IgAV-N patients (Table 2). The IgD, IgG and IgM serum concentrations and the Igλ/Igκ light-chain ratios were similar between IgAV patients and controls (Table 3).

**Table 3:** Biomarkers in the circulation and urine of pediatric patients and controls at the time of purpura rash (day 1).

	Controls	IgAV-woN	IgAV-N
**Immunoglobulins in serum**			
**IgA** (g/L)	1.0±0.2	2.7±0.2 ^**a**^	2.4±0.2 ^**b**^
**IgD** (mg/L)	17.0±6.0	23.1±4.7	27.1±5.5
**IgE** (μg/L)	49.0±13.2	149.0±25.2 ^**c**^	164.0±27.0 ^**b**^
**IgG** (g/L)	9.0±0.4	10.5±0.7	8.4±0.7
**IgM** (g/L)	1.0±0.1	1.3±0.1	1.4±0.1
**Igλ/Igκ ratio**	0.4±0.1	0.5±0.0	0.5±0.0
**Immunoglobulins in urine**			
**IgA/Cr** (g/mmol)	0.1±0.0	0.1±0.0	1.4±0.3 ^**b**^^,^ ^**d**^
**IgD/Cr** (mg/mmol)	nd	nd	nd
**IgE/Cr** (μg/mmol)	nd	nd	nd
**IgG/Cr** (g/mmol)	0.7±0.1	0.4±0.0	4.9±1.2 ^**e**^
**IgM/Cr** (g/mmol)	0.0±0.0	0.0±0.0	0.2±0.2 ^**b**^^,^ ^**d**^
**Igλ/Igκ ratio**	0.7±0.2	0.6±0.2	1.5±0.4 ^**e**^
**Cytokines in plasma**			
**IL-1β** (pg/mL)	1.0±0.3	8.3±2.5 ^**a**^^,^ ^**d**^	1.9±0.4
**IL-6** (pg/mL)	1.0±0.5	5.1±0.7 ^**a**^^,^ ^**e**^	3.6±0.6 ^**b**^
**IL-8** (pg/mL)	10.0±2.6	95.8±25.4 ^**a**^^,^ ^**e**^	16.5±3.0
**IL-10** (pg/mL)	3.0±0.7	1.7±0.8	1.6±0.3
**IL-12p70** (pg/mL)	1.0±0.5	2.9±0.8	2.1±0.4
**TNF-α** (pg/mL)	1.0±0.2	1.2±0.3	1.1±0.4
**Cytokines in urine**			
**IL-1β/Cr** (μg/mmol)	1.0±0.4	1.3±0.6	3.8±1.0
**IL-6/Cr** (μg/mmol)	0.0±0.1	0.6±0.2	4.5±1.1 ^**b**^^,^ ^**e**^
**IL-8/Cr** (μg/mmol)	2.0±0.6	1.6±0.5	10.9±2.4 ^**b**^^,^ ^**e**^
**IL-10/Cr** (μg/mmol)	0.1±0.1	0.2±0.1	0.8±0.1 ^**e**^
**IL-12p70/Cr** (μg/mmol)	0.0±0.0	0.0±0.0	0.5±0.3
**TNF-α/Cr** (μg/mmol)	0.0±0.0	0.0±0.0	0.3±0.2
**NGAL**			
**NGAL in plasma** (ng/ml)	118.0±18.5	297.1±35.7 ^**a**^	240.3±30.3 ^**b**^
**NGAL/Cr in urine** (μg/mmol)	6.1±1.1	2.5±0.6	7.0±1.5

Values are represented as the mean ± sem. Mann-Whitney U test.

^**a**^ controls vs IgAV-woN, P<0.0001

^**b**^ controls vs IgAV-N, P<0.0001

^**c**^ controls vs IgAV-woN, P<0.01

^**d**^ IgAV-woN vs IgAV-N, P<0.0001

^**e**^ IgAV-woN vs IgAV-N, P<0.01.

Abbreviations: Cr = creatinine, nd = not detected, NGAL = neutrophil gelatinase associated lipocalin

**Fig 1:**
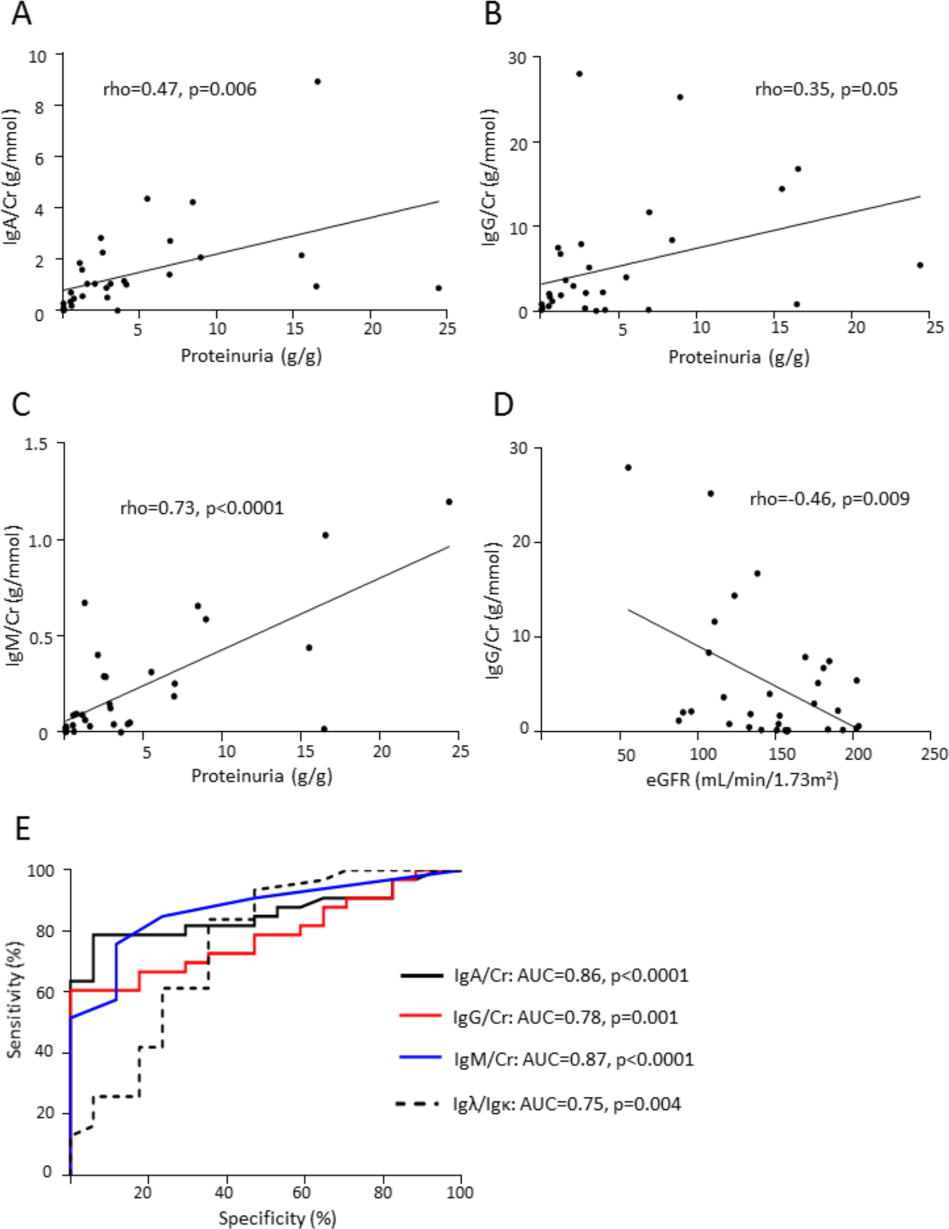
Urinary immunoglobulins and correlation with clinical parameters. Correlations between urinary IgA (A), IgG (B), IgM (C) and proteinuria in IgAV-N patients. (D) Correlations between urinary IgG and eGFR in IgAV-N patients. (E) Receiver operating characteristic (ROC) curves of the urinary concentrations of IgA, IgM, IgG and Igλ/Igκ ratio comparing the IgAV-woN and IgAV-N group.

IgD and IgE were not detected by ELISA in urine. IgA, IgG and IgM concentrations were increased in IgAV-N patients compared with the controls (p<0.0001) and with IgAV-woN patients (p<0.0001) (Table 3). The Igλ/Igκ ratio in urine samples was also increased for IgA-N patients compared with the healthy controls and IgA-woN patients (p<0.0001; Table 3). IgA, IgG and IgM urine concentrations were positively correlated with proteinuria (rho = 0.47, p = 0.006; rho = 0.35, p = 0.05; rho = 0.73, p<0.0001, respectively, Fig 1A, 1B and 1C). Only urinary IgG was negatively correlated with eGFR (rho = -0.46, p = 0.009, Fig 1D). Moreover, IgA, IgG, and IgM levels and the Igλ/Igκ ratio could accurately identify patients with nephritis (AUC = 0.86, confidence interval (CI) = 0.75–0.96, p<0.0001; AUC = 0.78, CI = 0.66–0.90, p = 0.001; AUC = 0.87, CI = 0.77–0.97, p<0.0001; and AUC = 0.75, CI = 0.59–0.90, p = 0.004, respectively; Fig 1E).

### IgA1 glycosylation and IgA complexes

**Fig 2:**
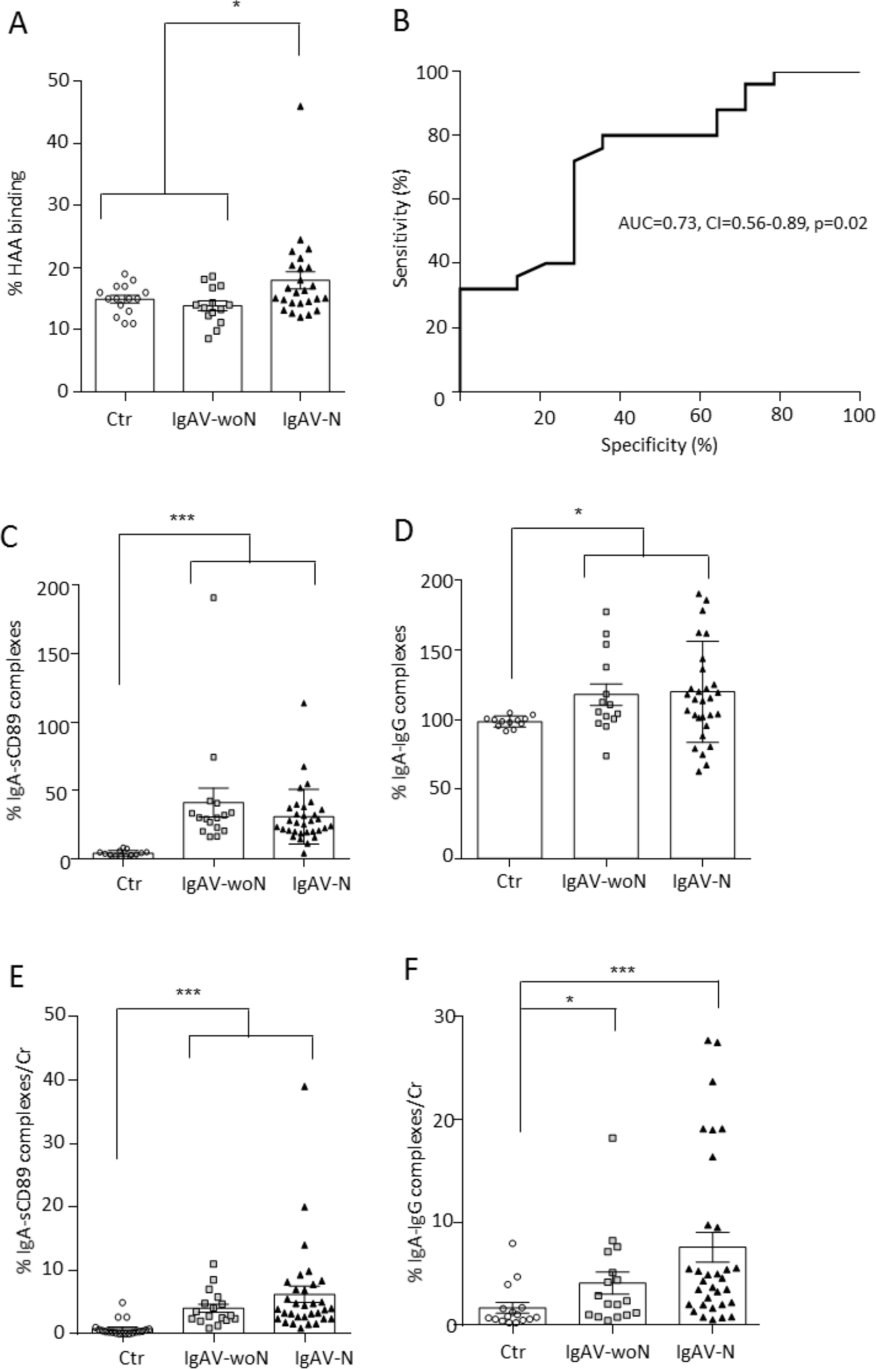
Markers of IgA nephropathy in IgAV. (A) Percentages of HAA binding on circulating IgA corresponding to Gd-IgA1 levels. (B) ROC curve of the serum Gd-IgA1 levels comparing the IgAV-woN and IgAV-N group. (C) Percentages of IgA-sCD89 complex level in patient sera. (D) Percentages of IgA-IgG complex level in patient sera. (E) Percentages of IgA-sCD89 complex level in patient urine. (F) Percentages of IgA-IgG complex level in patient urine.

After the examination of the IgA quantities, we assessed the levels of IgA1 glycosylation in IgAV patients: the O-glycosylation of IgA1. Circulating galactose-deficient IgA1 (GD-IgA1) was more elevated in IgAV-N patients compared to controls and in IgAV-woN patients (Fig 2A), as previously described [7], and this feature could be used to identify patients with nephritis (AUC = 0.73, CI = 0.56–0.89, p = 0.02; Fig 2B). No correlations were found with proteinuria or eGFR.

**Fig 3:**
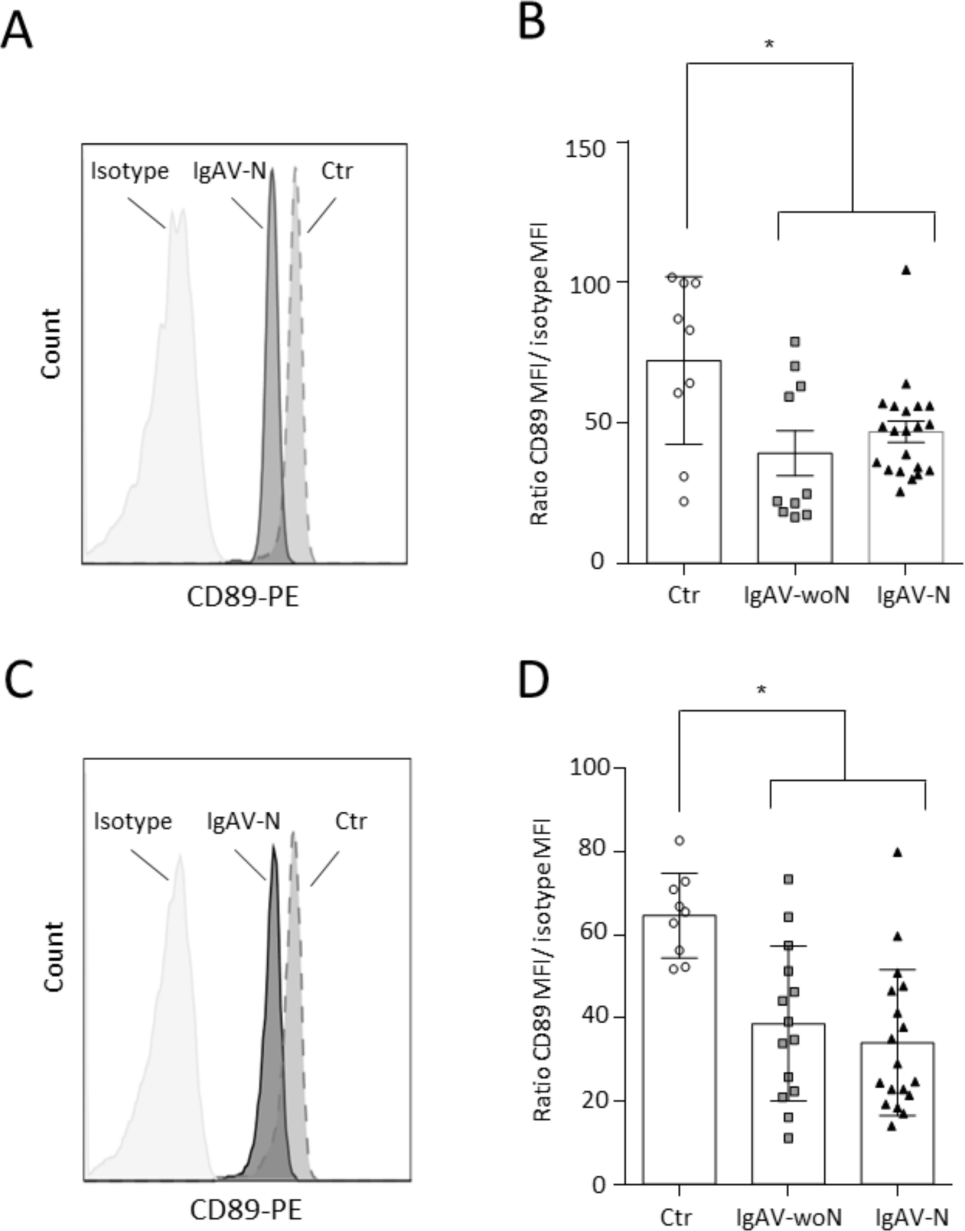
CD89 expression on blood granulocytes and monocytes. (A) Representative histogram of CD89 expression on granulocytes. (B) Ratio of the CD89 mean fluorescence intensity (MFI) / isotype MFI on blood granulocytes. (C) Representative histogram of CD89 expression on monocytes. (D) Ratio of the CD89 mean fluorescence intensity (MFI) / isotype MFI on blood monocytes.

Other markers of IgA nephropathy [12] were examined in IgAV patients. The levels of circulating IgA complexes (IgA-sCD89 and IgA-IgG) were increased in all IgAV patients compared with the controls (p<0.0001; Fig 2C and 2D, respectively). In urine samples, these complex levels were also increased in all IgAV patients compared with the healthy controls and IgAV-woN patients (p<0.001 and p<0.01 respectively; Fig 2E and 2F). IgAV-N patients exhibited more excretion of IgA complexes compared to IgAV-woN, but this increase was not statistically significant. Since the increase of circulating sCD89 in IgAV patients could be due to an increase of CD89 shedding at the cell surface of immune cells, the expression of CD89 was assessed on fresh blood samples by flow cytometry. There was a significantly decreased expression of CD89 on the surface of circulating granulocytes (p = 0.007; Fig 3A and 3B) and monocytes (p = 0.0006; Fig 3C and 3D).

### Cytokines

We then measured the concentrations of pro-inflammatory cytokines in plasma using CBA assays. IL-1β, IL-6 and IL-8 concentrations were higher in the IgAV group than in the control group (Table 2). The serum concentrations IL-12p70, TNF-α and the anti-inflammatory IL-10 were similar among IgAV patients and controls (Table 2).

**Fig 4:**
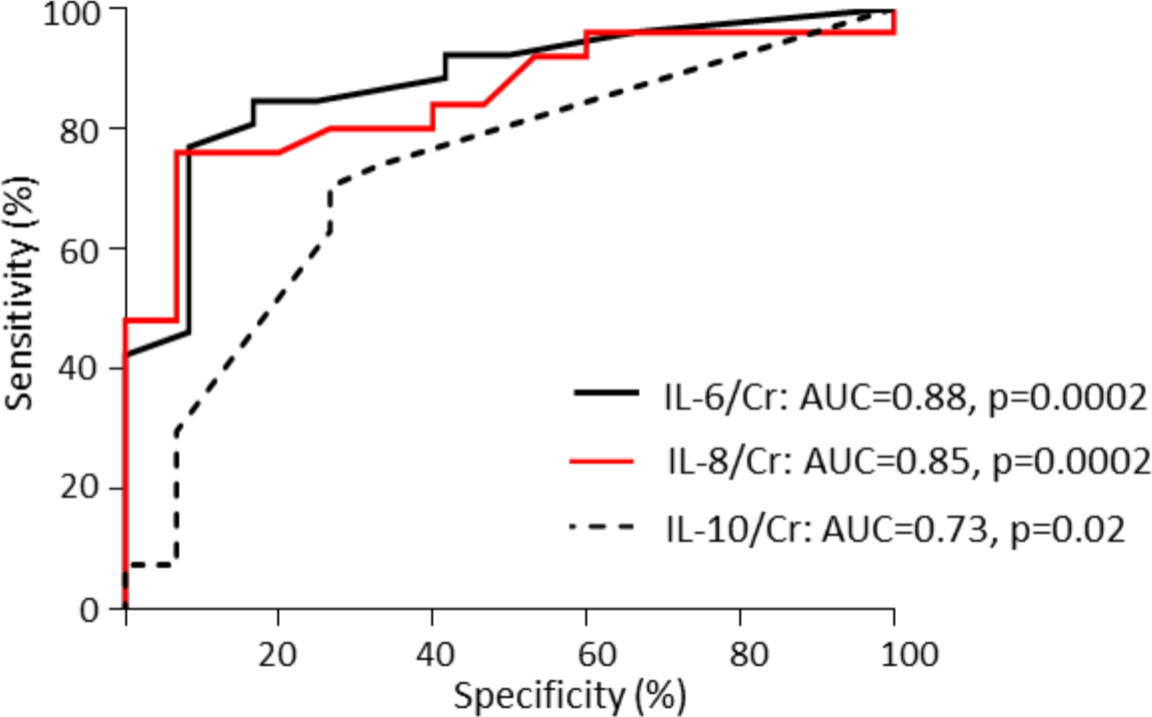
ROC curves of urinary cytokines comparing the IgAV-woN and IgAV-N groups.

IgAV nephritis in children was associated with the urinary excretion of IL-6, IL-8 and IL-10. Their concentrations were significantly higher in IgAV-N patients relative to healthy controls and IgAV-woN patients (p<0.0001; Table 2). Moreover, IL-6, IL-8 and IL-10 concentrations could accurately identify patients with nephritis (IL-6: AUC = 0.88, CI = 0.76–0.99, p = 0.0002; IL-8: AUC = 0.85, CI = 0.73–0.97, p = 0.0002; IL-10: AUC = 0.73, CI = 0.56–0.89, p = 0.02; Fig 4).

### NGAL

The NGAL plasma concentration was increased in IgAV pediatric patients compared with the matched controls (p<0.0001, Table 2) with no difference between IgAV-woN and IgAV-N patients. In urine, NGAL was not a discriminating biomarker of nephritis for children (Table 2).

## Discussion

In this prospective, multicenter study of a cohort of IgAV children, all of whom were included at the initial presentation of the disease, we described the clinical and biological features of the disease. In addition, we identified noninvasive biomarkers that could be used to safely diagnose IgAV nephritis in children. These results should be confirmed in a larger pediatric cohort and with a longer follow-up.

### Immunoglobulins in IgAV

IgA deposition in the different organs led to the study of immunoglobulin production in IgAV. Previous studies showed increased IgA [6,13,14] and IgE [6,13,15] serum concentrations in IgAV children and one described an increase in IgD in 39 patients [16]. We confirmed these results for IgA and IgE, but not for IgD, in our pediatric cohort. These elevations did not accurately discriminate patients with nephritis from those without. Urinary IgA, IgM and IgG were more indicative of nephritis in our cohort. Moreover, other groups have studied the presence of immunoglobulins in organ deposits. IgM has been associated with IgA skin deposits in children [17] and nephritis in adults [18–20]. IgG deposition in the kidney was associated with the nephritis outcome in adult IgAV patients [20].

### Hypogalactosylation of IgA1

The decrease of galactosylation in the hinge region of IgA1 (Gd-IgA1) molecules has been proposed to be the first pathological hit for kidney IgA1 deposition [7,21]. We confirmed in our pediatric IgAV cohort that the increase in serum Gd-IgA1 level was only associated with nephritis. Moreover, it has been described that in IgA-N, the recognition of this IgA1 hinge region neo-epitope, due to hypogalactosylation by IgA or IgG antibodies leads to the generation of immune complexes in the circulation (i.e., Gd-IgA1-IgA, Gd-IgA1-IgG) [22]. Here, we found that the level of IgA-IgG complexes was increased in the circulation of all pediatric patients, as previously described [23]. In contrast, in urine, these IgA complex levels were only associated with nephritis.

### IgA receptors

The receptor FcαRI or CD89 binds to IgA followed by cleavage of the CD89 extracellular domain and the release of nephrotoxic IgA-sCD89 complexes. In IgA nephropathy (IgA-N), these immune complexes are trapped in the glomerular mesangium by the mesangial transferrin receptor, which serves as an IgA1-sCD89 receptor [24]. The same pathological phenomenon also seems to occur in IgAV. In children, the circulating IgA-sCD89 complex level was increased in all IgAV patients and was associated with the decreased expression of CD89 at the cell surface of circulating granulocytes and monocytes. IgAV-N patients exhibited a higher level of these complexes in their urine. Adult patients with progressive IgA nephropathy had lower serum levels of IgA-sCD89 complexes than did patients with a good prognosis [25], as did recurrent IgA-N patients after grafting compared to non-recurrent IgA-N patients [26].

### Cytokines in IgAV

Concerning pro-inflammatory cytokines, we found elevated levels of IL-1β, IL-6 and IL-8 in the serum of all patients, with or without nephritis, compared to the controls. Urine IL-6, IL-8, and IL-10 concentrations distinguished IgAV with or without nephritis. Those cytokines have been implicated in IgA-N and IgAV with nephritis, particularly in mesangial cell activation, proliferation, crescent formation and glomerulosclerosis. Higher urine IL-6 levels at the time of IgA-N diagnosis increased the risk of progression in 59 patients followed for 8 years [27]. Increased serum IL-6 has been associated with the acute phase of IgAV [28]. More recently, serum IL-6 and IL-8 levels were found to be significantly increased in cases with renal involvement [29]. Circulating tumor necrosis factor receptor levels have been shown to be correlated with interstitial fibrosis and tubular atrophy in kidney biopsies, regardless of renal function; in addition, they were predictive of renal progression in 347 IgA-N cases confirmed by biopsy, indicating a role of the TNF pathway in this type of glomerulonephritis, which has been demonstrated in other chronic kidney diseases, such as diabetes or lupus nephritis [30]. Moreover, in small cohorts of IgAV patients, the serum level of TNFα was higher in IgAV-N than in IgAV-woN patients [31] and higher in active-phase IgAV than in the controls [13,32].

### NGAL as a marker of kidney injury

Neutrophil gelatinase-associated lipocalin (NGAL) is a 25-kDa acute-phase protein that was originally purified from human neutrophils and is markedly induced in injured epithelial cells. When tubular damage occurs, NGAL mRNA expression is significantly upregulated in the distal nephron segment to promote cellular proliferation and differentiation [33,34]. The NGAL protein is released into the circulation, freely filtered by the glomerulus and reabsorbed in the proximal tubules. Thus, following tubular damage, both serum and urine NGAL expression levels are increased. Many studies have been published on the predictive value of NGAL in acute renal injury (e.g., cardiac surgery, contrast-induced nephropathy, kidney transplantation). In IgAV patients, urinary NGAL seems to distinguish adult patients with or without nephritis [35] similarly to children [36]. An increased serum NGAL concentration was present in both IgAV-N and IgAV-woN children in our study. There was a tendency for increased urinary NGAL in IgAV-N compared to IgAV-woN patients.

### Conclusions

This prospective cohort study, with pediatric patients who were included at the onset of the disease, identified biomarkers that are able to segregate patients initially with or without nephritis. The serum levels of Gd-IgA1 and urinary levels of IgA, IgG, IgM, IL-6, IL-8, and IL-10 complexes showed an accurate predictive performance in the identification of IgAV patients with renal involvement at the time of diagnosis.
